# Assessing Quality Gaps and Clinician Perspectives on AI Integration in Colorectal Cancer NGS Pathways

**DOI:** 10.3390/curroncol33090540

**Published:** 2026-09-09

**Authors:** Luxiga Thanabalachandran, Darya Ali, Avery Newman-Simmons, Claire Norman, Daniel Jafari, Tamara Grahovac, Aaron Pollett, Weei-Yuarn Huang, Lina Chen, Shaqil Kassam, Carly C. Barron, Suneil Khanna, Kristin Wright, Stephanie Snow, Ron Burkes, Tao Wang, Andrea Grin, Taylor Moffat, Yuchen Li

**Affiliations:** 1 School of Medicine, Queen’s University, Kingston, ON K7L 3N6, Canada; claire.norman@kingstonhsc.ca (C.N.); tamara.grahovac@kingstonhsc.ca (T.G.); 2 Department of Pathology and Molecular Medicine, Queen’s University, Kingston, ON K7L 3N6, Canada; darya.ali@kingstonhsc.ca (D.A.); avery.newmansimmons1@kingstonhsc.ca (A.N.-S.); tao.wang@kingstonhsc.ca (T.W.);; 3 Department of Biomedical and Molecular Sciences, Queen’s University, Kingston, ON K7L 3N6, Canada; daniel.jafari@kingstonhsc.ca; 4 Department of Laboratory Medicine & Pathobiology, Mount Sinai Hospital, Toronto, ON M5G 1X5, Canada; aaron.pollett@sinaihealth.ca; 5 Department of Laboratory Medicine & Pathobiology, Sunnybrook Health Science Centre, Toronto, ON M4N 3M5, Canada; weeiyuarn.huang@sunnybrook.ca (W.-Y.H.); lina.chen@sunnybrook.ca (L.C.); 6 Southlake Health, Newmarket, ON L3Y 2P9, Canada; skassam@southlake.ca; 7 Princess Margaret Cancer Centre, Toronto, ON M5G 2C4, Canada; carly.barron@uhn.ca; 8 St. Michael’s Hospital, Unity Health Toronto, Toronto, ON M5B 1W8, Canada; suneil.khanna@unityhealth.to; 9 Department of Oncology, Queen’s University, Kingston, ON K7L 3N6, Canada; kristin.wright@kingstonhsc.ca (K.W.); taylor.moffat@kingstonhsc.ca (T.M.); yuchen.li@kingstonhsc.ca (Y.L.); 10 Cancer Centre of Southeastern Ontario, Kingston Health Sciences Centre, Kingston, ON K7L 2V7, Canada; 11 Division of Medical Oncology, QEII Health Sciences Centre, Halifax, NS B3H 3A7, Canada; stephanie.snow@nshealth.ca; 12 Division of Hematology Oncology, Sinai Health, Toronto, ON M5G 1X5, Canada; ron.burkes@sinaihealth.ca

**Keywords:** colorectal cancer, next-generation sequencing, molecular testing, quality improvement, care pathways, digital health, artificial intelligence, pathology

## Abstract

Next-generation sequencing (NGS) is integral to colorectal cancer (CRC) care, but its value depends on results being available when treatment decisions are made. We conducted a qualitative study to identify quality gaps in CRC NGS pathways and assess perspectives on digital and artificial intelligence (AI)-enabled workflow support. Semi-structured interviews were conducted with 22 medical oncologists and pathologists from 14 Ontario hospitals, and transcripts were analyzed thematically. Five themes emerged: (1) clinical indications and test ordering, where initiation was inconsistent because of unclear reflex-testing criteria, reliance on upstream clinicians, staging uncertainty, evolving indications, and funding constraints; (2) tissue, pathology, and laboratory workflow, where participants attributed bottlenecks and turnaround variability to send-out testing, staffing, infrastructure, and capacity; (3) interdisciplinary communication and tracking, where informal communication, unclear ownership, and manual tracking highlighted the need for closed-loop confirmation and alerts; (4) reporting and clinical integration, where report clarity, interpretability, and timing affected use; and (5) electronic health record integration and AI integration, where fragmented systems and limited interoperability delayed result access. Participants supported clinician-supervised digital or AI-enabled tools for case identification, triage, tracking, and overdue-result alerts. CRC NGS optimization requires standardized reflex criteria, clearer ownership, closed-loop tracking, reliable routing, structured reporting, and interoperable infrastructure.

## Simple Summary

For people with advanced colorectal cancer, choosing the most effective treatment depends on timely genetic testing of the tumour. When these results are delayed or hard to access, treatment may be postponed or started before the best targeted option is known. Because this testing involves many steps, from biopsy and pathology review through laboratory processing, reporting, and decision-making, delays and inefficiencies can arise. We interviewed 22 medical oncologists and pathologists from 14 hospitals across Ontario, Canada, to find where delays, omissions, and gaps occur, and to gauge their perspectives on wider use of digital tools and artificial intelligence in testing. Participants described vulnerabilities in how testing is started, tracked, owned, reported, and linked to electronic health records. They were optimistic that validated, clinician-supervised digital and artificial intelligence tools could help identify cases, track progress, and flag overdue results. These findings highlight practical ways to make testing more reliable and equitable.

## 1. Introduction

Colorectal cancer (CRC) is among the most commonly diagnosed malignancies worldwide and remains a leading cause of cancer-related mortality [1]. In 2024, more than 2 million new CRC cases and approximately 918,000 CRC deaths were estimated globally [2]. In Canada, approximately 25,200 new CRC cases and 9400 CRC deaths were projected in 2024, making CRC the fourth most commonly diagnosed cancer and the second leading cause of cancer-related death nationally [3]. Population-based screening and treatment personalization through molecularly guided therapy have become increasingly important components of CRC care. In advanced CRC specifically, advances in biomarker-directed therapies have made timely molecular characterization using next-generation sequencing (NGS) increasingly essential for treatment selection. Contemporary Canadian and American practice guidelines therefore recommend NGS testing at the time of metastatic diagnosis, including RAS (KRAS and NRAS), BRAF V600E, and HER2/ERBB2 alterations [4,5]. Landmark targeted-therapy studies, including KEYNOTE-177, CodeBreaK 300, BREAKWATER, and DESTINY-CRC02, illustrate how treatment selection increasingly depends on molecular results being available early in the care pathway [6,7,8,9].

The clinical value of molecular profiling depends on results being available at the point of treatment decision-making. When NGS results are delayed, clinicians may need to begin non-targeted therapies before actionable alterations are known, potentially limiting or delaying access to biomarker-directed treatment and clinical trials. Although delays in cancer treatment more broadly have been associated with adverse patient outcomes [10], direct evidence that delays in NGS results themselves worsen survival in CRC remains limited. Yet, turnaround times vary substantially across institutions and testing models [11,12], reflecting a multistep pathway that spans biopsy, pathology review, test initiation, laboratory processing, reporting, result routing, and clinical action.

Despite the growing importance of molecular testing in CRC, few studies have examined how CRC NGS pathways function in routine practice, and fewer still have incorporated multidisciplinary perspectives, particularly those of pathologists, who initiate testing, process samples, and frequently interpret molecular results. Although limited prior research has demonstrated variation in reflex versus clinician-initiated testing, tissue handling, and turnaround time [13,14], the barriers and quality gaps within CRC-specific NGS workflows have not been systematically characterized. Reflex testing is proposed to reduce clinician-level variability and improve the likelihood that complete results are available by the first oncology consultation [15,16], yet implementation remains uneven. These pathways are also unlikely to be uniformly equitable: differences between academic and community settings, in-house versus send-out testing models, and evolving funding criteria may all shape which patients receive complete, timely profiling [17,18], and may widen existing inequities in access to precision oncology.

Artificial intelligence (AI) is increasingly being investigated across oncology, including radiologic and histopathologic image interpretation, molecular phenotype prediction, risk stratification, clinical-trial matching, natural-language processing, and clinical decision support [19,20]. More recently, multimodal and generative AI systems, including large language model (LLM)-based tools, have been evaluated for integrating clinical, imaging, pathology, and molecular information to support oncology decision-making [21]. However, clinical adoption remains heterogeneous, and prospective validation, workflow integration, human oversight, governance, and ongoing performance monitoring remain important requirements for safe implementation [22,23]. Less is known about how clinicians view the integration of these technologies into existing NGS workflows.

Accordingly, we conducted a qualitative study examining the perspectives of medical oncologists and pathologists across academic and community hospitals in Ontario, the most populous province in Canada. Our primary aim was to identify quality gaps across the CRC NGS pathway and define targeted quality improvement (QI) opportunities to improve timely, reliable, and equitable access to precision oncology. A secondary aim was to assess clinician perspectives on AI integration and to identify digital and electronic health record (EHR) infrastructure needs that could support more reliable NGS workflows.

An abstract based on preliminary findings from this study has been accepted for poster presentation at the 2026 ASCO Quality Care Symposium, to be held 16–17 October 2026, in Boston, Massachusetts, and will be published in the conference proceedings. The submitted manuscript provides a substantially expanded and complete report of the study, including the full methods, analyses, and results.

## 2. Materials and Methods

### 2.1. Study Design

We conducted a qualitative study using interpretive description, an applied qualitative methodology designed to generate clinically relevant insights for practice and quality improvement [24]. Semi-structured interviews were conducted with medical oncologists and pathologists involved in CRC NGS workflows across academic and community institutions. Interview data were analyzed using thematic analysis. Interpretive description served as the overarching methodological orientation, while thematic analysis was used as the analytic method. Interpretive description was selected because the study sought to generate clinically relevant, practice-oriented knowledge about how CRC NGS pathways function in routine care and how identified gaps might be addressed.

### 2.2. Ethics

Ethics approval was obtained from Queen’s University Health Sciences Research Ethics Board (HSREB Reference Number: 6045602). All participants provided written informed consent prior to participation.

### 2.3. Setting

The primary investigating site was the Cancer Centre of Southeastern Ontario, a regional academic centre in Kingston, Ontario, Canada. Participants were recruited from academic and community hospitals across Ontario to capture variation in institutional setting, geography, and local NGS testing models.

### 2.4. Sampling and Recruitment

We used purposeful maximum-variation sampling to recruit medical oncologists and pathologists with direct involvement in ordering, interpreting, reporting, or coordinating NGS testing for CRC. Maximum-variation was sought across professional roles and academic versus community settings. These characteristics were selected because they were expected to yield distinct and important perspectives on NGS workflow. Referral sampling was also used to identify additional eligible participants.

Eligible participants were invited by email. Of 59 individuals invited, 22 participated, including 9 medical oncologists and 13 pathologists from 14 institutions. Data collection and preliminary analysis occurred concurrently. The research team periodically reviewed the evolving codebook and thematic structure to determine whether subsequent interviews generated new codes or substantively altered existing themes. Recruitment continued until thematic saturation was reached after the 22nd interview. Saturation was assessed across the overall dataset rather than separately within academic/community or in-house/send-out subgroups.

### 2.5. Data Collection

Interviews were conducted between March and May 2026 by two pathology residents, a senior medical student, and two staff medical oncologists. A semi-structured interview guide (Appendix A) was developed based on the literature, the steps of the CRC NGS pathway, and multidisciplinary input from oncology and pathology team members. The guide explored test initiation and ordering, tissue acquisition and adequacy, laboratory workflow, turnaround time, result tracking, reporting and clinical integration, interdisciplinary communication, digital and electronic health record infrastructure, equity, and opportunities for quality improvement. AI-related interview questions were exploratory and were intended to understand clinicians’ perceptions of potential AI applications, integration, and safety considerations within CRC NGS workflow. The study did not apply a validated AI-readiness framework or comprehensively assess institutional technical, organizational, or operational readiness.

Interviews were conducted virtually using Microsoft Teams and lasted approximately 45 min. Interviews were audio-recorded and transcribed verbatim. Transcripts were de-identified before analysis.

### 2.6. Data Analysis

Interview transcripts were analyzed using thematic analysis [25] in MAXQDA (VERBI Software, Berlin, Germany), within a broader methodological framework of interpretive description. Coding was primarily inductive, allowing codes and themes to emerge from participants’ accounts, while remaining informed by the study objective of identifying quality gaps across the CRC NGS pathway.

Two researchers independently coded an initial subset of five transcripts and developed a preliminary codebook. Coding discrepancies were resolved through discussion, and the codebook was refined iteratively, as additional transcripts were reviewed. Codes were compared across professional roles and institutional settings. Once coding consistency was established, the remaining transcripts were coded using the refined codebook. The study team met regularly to review emerging codes, compare interpretations, and refine theme development. Analytic rigour was supported through investigator triangulation, iterative codebook refinement, and multidisciplinary team discussion. For each subtheme reported in Appendix A Table A1, the accompanying value (e.g., *n* = 18) denotes the number of participants who mentioned that subtheme in their interview. These counts are provided to indicate the distribution of topics across participants and should not be interpreted as estimates of prevalence or as a measure of the relative importance or strength of a theme. Theme development was based on explanatory and clinical relevance, consistency across professional and institutional contexts, and implications for the CRC NGS pathway rather than frequency alone.

### 2.7. Researcher Reflexivity

Interviews were conducted by two pathology residents, one senior medical student, and two staff medical oncologists. Throughout the analysis, the research team reflected on and discussed how members’ professional roles, clinical experiences, and assumptions might influence coding and theme development. Independent coding of an initial subset of five transcripts, followed by multidisciplinary review incorporating both oncology and pathology perspectives, was used to identify divergent interpretations and mitigate reliance on any single disciplinary perspective. Interviewers had no prior known relationships with the interviewed participants.

## 3. Results

**Figure 1:**
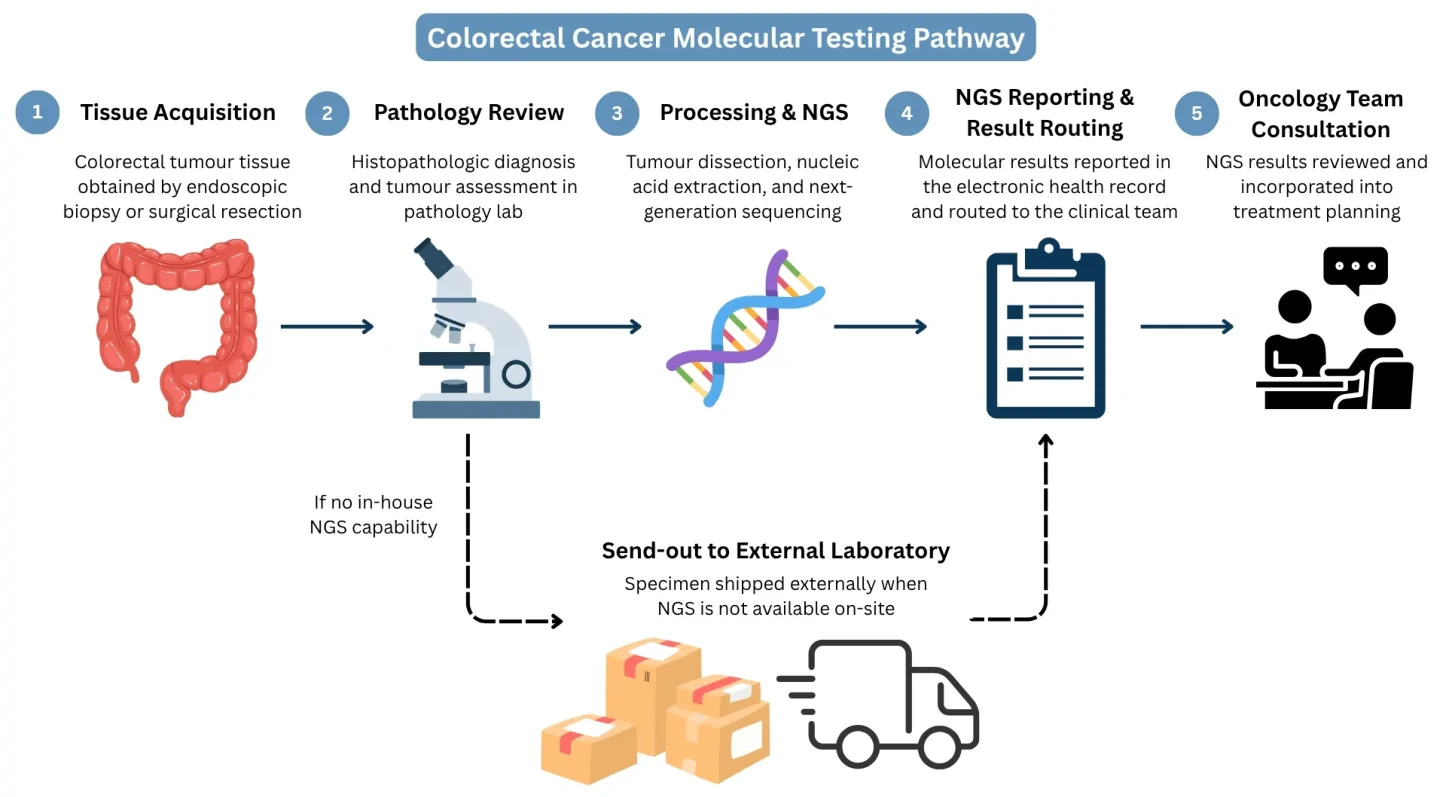
Overview of the colorectal cancer molecular testing pathway. Colorectal tumour tissue undergoes pathology review and NGS, either in-house or at an external laboratory. Results are reported in the EHR and routed to the clinical team to support treatment planning.

Twenty-two medical oncologists and pathologists from 14 academic and community institutions across Ontario, Canada participated (Table 1). Participants comprised 9 medical oncologists and 13 pathologists. Seventeen participants worked in settings where NGS was performed in-house and five relied on send-out testing or referral to a secondary centre. Analysis generated five themes spanning the CRC NGS pathway: test initiation, tissue and laboratory workflow, communication and tracking, reporting and clinical integration, and digital infrastructure. Illustrative quotations are provided in Appendix A Table A1. An overview of the CRC molecular testing pathway, including both in-house and send-out NGS testing models, is shown in Figure 1.

### 3.1. Theme 1: Clinical Indications, Patient Identification, and Test Ordering

Participants described NGS test initiation as a major point of vulnerability, as responsibility for identifying eligible patients was distributed across several clinical roles. In many settings, NGS was initiated only after the medical oncology consultation, even when the diagnostic biopsy had occurred weeks earlier. This delay often occurred when upstream clinicians involved in the process, such as gastroenterologists or surgeons performing endoscopic biopsies, or radiologists, did not indicate that advanced disease was suspected. This often leaves pathologists with insufficient clinical and staging information to determine whether to complete NGS at the time of case sign-out, and therefore leaves them to inconsistently initiate NGS beyond routine mismatch-repair (MMR) immunohistochemistry. Once a case had been signed out, there was often no reliable mechanism to revisit the specimen after subsequent imaging confirmed advanced disease. As a result, medical oncologists commonly discovered at the first consultation that molecular testing had not been initiated and had to request it then.

*“Some pathologists are better than others at initiating that reflex, but it is quite subjective, because you’re not necessarily provided with information up front. It does require a bit of investigation on your part.”*

(Pathologist, community centre)

Further, although several centres had reflex-testing protocols for advanced CRC, implementation was uneven and influenced by staffing, laboratory capacity, local practice norms, and funding criteria. Participants generally supported the clinical value of molecular profiling, but described uncertainty about when eligibility could be confidently established and who was responsible for initiating NGS testing or flagging eligible cases to pathologists.

Evolving indications further complicated ordering practices. Several participants anticipated broader use of molecular testing beyond metastatic disease, including earlier-stage CRC and emerging applications such as recurrence-risk stratification and minimal residual disease testing. However, expanding testing indications was viewed by some participants as potentially challenging with existing workflows, which are already strained by testing volume, costs, and administrative burden.

Funding coverage also consistently shaped NGS ordering decisions. Provincially defined funding criteria were described as necessary for stewardship, given the cost and resource requirements of NGS, but also as a source of variability when indications evolved faster than reimbursement pathways. Some laboratories performed testing based on funded indications due to test volume and capacity constraints, while others proceeded with testing outside formal criteria when clinicians felt it was clinically necessary.

Together, these findings suggest that delayed test initiation reflects the absence of clear frameworks for timely eligibility recognition, communication, and ownership across the early diagnostic pathway, rather than questions about the utility of NGS.

### 3.2. Theme 2: Tissue, Pathology, and Molecular Laboratory Workflow

Once testing was initiated, participants described molecular pathology laboratory capacity and tissue-handling workflows as the main determinants of turnaround time. Testing models varied across institutions, with some centres performing NGS in-house and others sending out specimens to other, often larger laboratories at academic centres. Send-out testing was sometimes associated with transport delays, referral-centre backlogs, and limited ability to monitor test status. Participants from smaller or rural community hospitals described send-out testing as necessary, but burdensome, and some expressed a desire to bring testing in-house to improve speed and communication.

Laboratory capacity emerged as a recurring structural constraint. Participants described shortages of molecular pathologists, molecular geneticists, and laboratory technologists, as well as delays related to reagent issues, instrument downtime, and queuing for extraction, sequencing, and sign-out. Several participants emphasized that local turnaround-time targets were inconsistently met and, in some cases, were perceived to be exceeded by several weeks or months.

*“Most places, other than a couple in Ontario, are well past that benchmark, but there’s really no auditing or repercussions.”*

(Medical oncologist, academic centre)

Participants also described process heterogeneity across sites. For example, approaches to tissue dissection ranged from pathologist-directed tumour circling and macrodissection to whole-slide scraping workflows. These local differences influenced both turnaround time and workload.

Thus, tissue and laboratory barriers were not limited to technical sequencing capacity, but reflected the cumulative effect of local workflow design, staffing, infrastructure, and handoffs between diagnostic and molecular pathology processes.

### 3.3. Theme 3: Interdisciplinary Communication and Test Tracking

The absence of a shared NGS tracking system created a coordination gap between test ordering, laboratory processing, and clinical action. Participants described communication as highly dependent on informal channels, including email, direct messaging, and individual follow-up. These workarounds were often effective when clinicians knew whom to contact, but they were not standardized, scalable, or reliable across institutions and care transitions.

Ownership of the NGS pathway was frequently unclear. Pathologists were often viewed as stewards of tissue and testing processes, but they did not always have the clinical information needed to determine eligibility. Medical oncologists, in turn, often assumed responsibility for ensuring that NGS testing was completed, because they depended on the results for treatment decisions. Surgeons, gastroenterology endoscopists and radiologists were seen as important upstream partners in identifying patients for NGS testing, but were often less engaged in downstream molecular testing, because they did not directly deliver biomarker-directed systemic therapies.

Participants commonly relied on manual tracking strategies, including personal lists, pending piles, notes, and repeated email follow-up. These manual systems compensated for gaps in institutional infrastructure but introduced vulnerability, particularly when clinical workload increased. Several participants described uncertainty about whether a test had been ordered, whether tissue had been sent, where the specimen was in the testing process, or when results were expected.

*“Every pathologist has their own system in terms of how they decide to track it. I have my pending pile, and then I go through it periodically to make sure things are moving through.”*

(Pathologist, community centre)

A consistent improvement idea proposed by several participants was the implementation of a closed-loop confirmation system that tracks and documents each step of the NGS pathway, from order placement and specimen receipt through processing, pathologist sign-out, and result release. Participants wanted EHR work-queue functionality that could display real-time NGS status, flag overdue results, and ensure that completed reports were routed to the clinician responsible for treatment decisions. Without such systems, a test could be completed, but remain clinically invisible if the result was sent only to the original ordering proceduralist rather than the treating medical oncology team.

### 3.4. Theme 4: Reporting, Interpretability, and Clinical Integration

Participants described NGS reports as clinically valuable but variable in format, content, and usability. Laboratories differed in how results were organized and whether therapeutic implications were clearly stated. Although participants agreed on the relevance of core biomarkers such as RAS, BRAF, HER2/ERBB2, and PIK3CA, report structure influenced how easily actionable findings could be identified and applied.

Improving NGS report usability was a recurring priority, with participants emphasizing that molecular results should be presented in a simple, accessible, and readily interpretable format to support accurate and efficient treatment planning, particularly amid increasing patient volumes and clinical demands. Participants valued reports that placed actionable findings at the beginning, used clear tiering of alterations, and linked results to therapeutic relevance. In contrast, lengthy, fragmented, or addendum-heavy reports were perceived as obscuring clinically important findings. This was particularly concerning when results were spread across multiple documents or were not integrated into the main pathology report.

The need for interpretive support varied by participant and case complexity. Most medical oncologists felt comfortable interpreting common CRC NGS results, whereas more complex and/or less frequently encountered molecular alterations, including variants of uncertain significance (VUS), may require further consultation with pathologists or discussion at molecular tumour boards. Participants viewed these supports as valuable but not universally available.

The most consequential clinical issue was timing. Participants repeatedly noted that NGS results were often unavailable at the first medical oncology consultation. In these cases, medical oncologists sometimes had to begin treatment before complete molecular information was available or delay treatment until results were received, with potential implications for first-line treatment selection, access to targeted therapies, clinical trial eligibility, and patient counselling.

*“Especially a first-line clinical trial looking at biomarker-driven therapy. The question is, if we can’t get the NGS quickly, then how can we run that trial in our center?”*

(Medical oncologist, academic centre)

### 3.5. Theme 5: EHR Integration, Digital Infrastructure, and Clinician Perspectives on AI Integration

Participants identified a lack of comprehensive digital infrastructure supporting the CRC NGS pathway as a key challenge. These participants described persistent reliance on paper forms, faxed results, scanned documents, and manual data entry. These processes were viewed as inefficient, but often remained embedded in local workflows because of EHR system limitations, perceived reliability, and the absence of integrated alternatives. As a result, molecular testing information could be difficult to locate even when testing had been completed.

Fragmented EHR visibility for NGS results was also a major barrier to timely result access. Participants described NGS results that were stored outside the main pathology report, housed in separate laboratory systems, uploaded as scanned documents, or unavailable across different institutions. Limited interoperability made it difficult to determine whether testing had been previously performed, particularly when patients moved between community hospitals, academic centres, and referral laboratories. In some cases, this contributed to duplicate testing or delayed treatment planning.

Participants identified digital tracking and automated notification as practical near-term solutions. Desired features included visibility into pending NGS orders, specimen status, expected turnaround time, overdue-result alerts, and automatic notification when results became available. These functions represent workflow automation and EHR optimization rather than AI.

Participants also saw potential for AI to support case identification, triage, and reflex initiation. Suggested applications included scanning clinical histories, pathology reports, or EHR data to identify patients who might meet testing criteria; flagging cases in which NGS should be considered; and monitoring whether expected results had been returned.

*“Making sure it’s being ordered and initiated as early as possible for appropriate indications is one way that AI could definitely help.”*

(Pathologist, community centre)

However, enthusiasm for AI was consistently followed by concerns about privacy, accuracy, generalizability, accountability, and the need for ongoing audit. Participants emphasized that AI should support, rather than replace, clinician judgment and that responsibility for final decisions should remain with the clinical team.

Participants therefore identified two complementary approaches to pathway improvement: conventional digital infrastructure to automate deterministic workflow tasks, and potential AI-enabled tools to support interpretation, classification, or prioritization of clinical information. Participants emphasized that AI-enabled tools should be implemented as validated, human-supervised systems, and designed to address concrete workflow gaps rather than adding another layer of fragmented technology.

**Figure 2:**
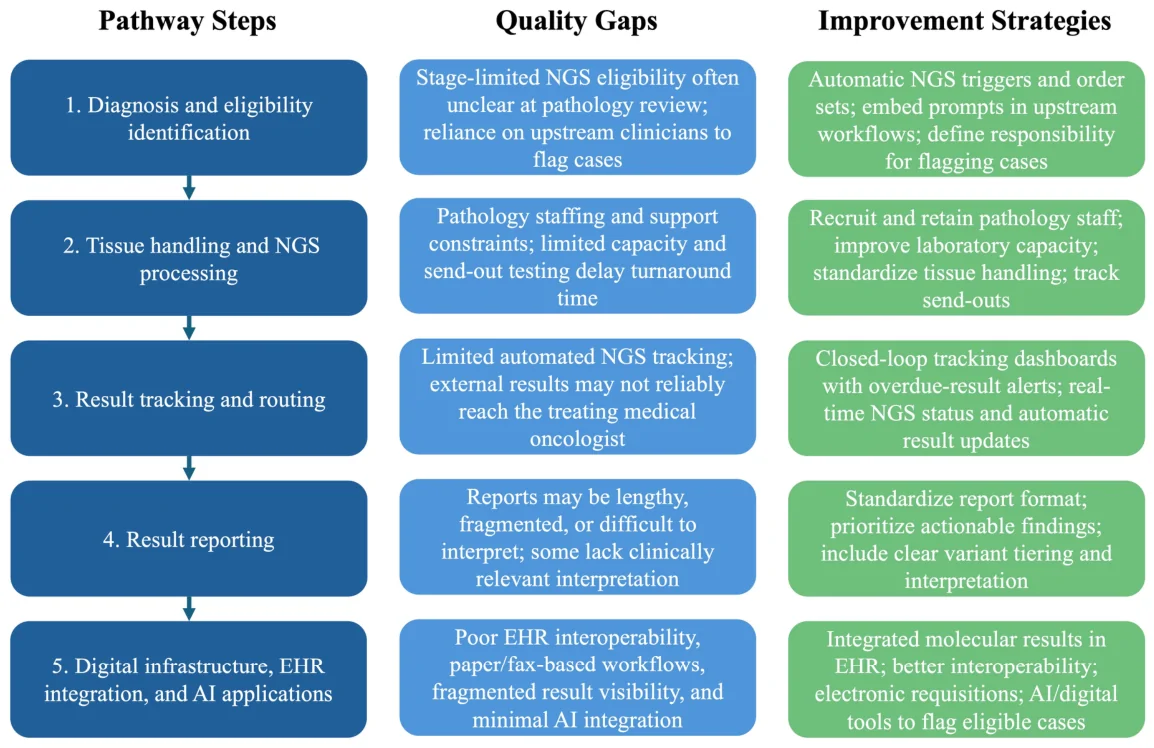
Quality gaps across the colorectal cancer next-generation sequencing testing pathway. The CRC NGS pathway spans biopsy and diagnosis, test eligibility identification, NGS ordering, tissue processing, result reporting, result routing, and oncology treatment decision-making.

Across themes, participants identified several modifiable quality gaps and potential improvement strategies. These included stage-appropriate reflex-testing criteria, clearer assignment of responsibility for ordering and tracking, closed-loop result management, reliable routing of results to medical oncology teams, standardized molecular reporting, integrated EHR visibility, and human-in-the-loop digital or AI-enabled tools. These opportunities are summarized in Figure 2 and Table 2.

## 4. Discussion

Timely access to molecular testing is increasingly important across many cancer types, including CRC, as treatment decisions become more personalized and biomarker-driven. Despite this shift, few studies have examined how NGS workflows function specifically in CRC, and fewer still have comprehensively captured the perspectives of both medical oncologists and pathologists. In this multi-institutional qualitative study, clinicians described CRC NGS as a pathway-level process that varied across institutions, was often insufficiently streamlined, and was sometimes hindered by poor interdisciplinary communication and fragmented EHR and digital integration. The central issue was not lack of support for molecular testing, but the absence of reliable, comprehensive systems to identify eligible patients early, assign ownership, track testing status, route completed results, and present findings in a clinically usable format. Framed this way, delays in CRC NGS are better understood as potentially modifiable gaps in coordination and infrastructure rather than as laboratory turnaround-time problems alone. By characterizing coordination between diagnosis, molecular testing, result routing, and treatment decision-making across academic and community hospitals in Ontario, Canada’s most populous province and part of a publicly funded health care system, this study extends prior work focused primarily on testing rates, biomarker completeness, and turnaround times [11,13,14,15].

The most actionable quality gaps identified in the NGS pathway were concentrated at points where formal processes, responsibilities, and tracking mechanisms were lacking.

At diagnosis and eligibility identification, metastatic status was often unknown at biopsy, and pathologists did not always have the clinical information needed to trigger reflex testing. At the ordering stage, responsibility could be distributed across upstream clinicians, including gastroenterology and surgical endoscopists, radiologists, pathologists, and medical oncologists, without a clear accountability structure. During tissue handling and NGS processing, delays were driven by send-out testing, local workflow design, staffing, instrument and reagent issues, batching, and sign-out workload. After testing was ordered, manual tracking and fragmented result routing created uncertainty about whether NGS had been ordered, received, processed, finalized, and reviewed by the responsible medical oncology team.

These findings support a practical QI agenda aligned with the pathway map (Figure 2 and Table 2): define stage-appropriate reflex-testing criteria and funding pathways; embed prompts or order sets into upstream clinical and pathology workflows; assign responsibility for tracking and result review; create closed-loop dashboards with overdue-result alerts; route completed reports to the responsible medical oncology team; and standardize report formats so actionable findings and therapeutic implications are visible at the point of care. More broadly, these findings may also inform advocacy by national organizations, such as the Canadian Pathology Quality Assurance (CPQA) [26], for improvements in molecular testing pathways beyond Ontario and across all cancer types.

Reporting and clinical integration represented additional opportunities for improvement. Participants preferred reports that placed actionable findings first, used tiered interpretation, and clearly linked molecular results to therapeutic implications. Conversely, lengthy or fragmented reports, especially those distributed across addenda or external documents, were perceived as increasing the risk that clinically important results would be missed. These findings align with broader evidence supporting synoptic and structured reporting to improve completeness, clarity, and interoperability [27]. For CRC NGS, report standardization should be viewed not only as a documentation improvement, but as a safety intervention that helps ensure biomarker information is available, visible, and interpretable when treatment decisions are made. At the national level, these findings could inform the development of a multidisciplinary white paper, incorporating perspectives from pathology and oncology, to establish guidance on the structure, content, and presentation of molecular reports.

Perhaps the most clinically significant consequence of these pathway gaps was the recurrent absence of NGS results at key decision points, including the initial medical oncology consultation and initiation of first-line therapy. Participants described situations in which clinicians initiated non-targeted therapy while awaiting results, with downstream implications for treatment selection, clinical trial eligibility, and the ability to use targeted options at the most appropriate time. This observation connects the workflow barriers identified in this study to broader evidence that delays in cancer care can adversely affect outcomes [10]. However, this broader evidence does not establish that delays in NGS results themselves worsen survival in CRC, and the present study did not evaluate patient outcomes associated specifically with NGS turnaround time. It also supports prioritizing upstream interventions that move testing earlier in the pathway, reduce preventable waiting time, and make result delivery more reliable before first-line treatment decisions are finalized.

These challenges are likely to become more pronounced as biomarker testing expands beyond advanced CRC. Participants anticipated increasing requests for testing in earlier-stage disease, including emerging recurrence-prevention and minimal residual disease applications. The ALASCCA trial and evolving interest in PIK3CA-pathway testing illustrate how new evidence can rapidly expand molecular testing from a metastatic-treatment tool to one that also informs risk stratification and adjuvant decision-making [28]. However, these findings do not establish the clinical utility or cost-effectiveness of broad comprehensive NGS testing across all patients with early-stage CRC. Without more standardized initiation criteria, tracking infrastructure, and laboratory capacity, expanding indications may amplify the same inefficiencies described in this study and widen variation between institutions.

Although established genomic biomarkers are commonly used for treatment selection, emerging non-genomic biomarkers are also being investigated in CRC. Recent studies have associated lower serum cholinesterase levels with poorer survival, while butyrylcholinesterase (BCHE) expression has been associated with pathologic stage, progression-free survival, and immune-cell infiltration [29,30]. However, these findings remain preliminary, and BCHE is not currently an established component of routine CRC molecular or NGS testing.

In addition to examining the CRC NGS pathway, our study explored the digital infrastructure supporting the pathway, participants’ perception of AI, and readiness for its use. Participants identified both conventional digital infrastructure improvements and potential AI-enabled applications as potential facilitators of pathway reliability. Participants described interest in conventional digital tools that could provide real-time order status, automated result notification, overdue-result alerts, integrated molecular result displays, and automatic routing to the responsible medical oncology team. They also identified potential AI-supported applications, including case identification, reflex-testing prompts, missing-order detection, overdue-result monitoring, and clinical trial matching. These applications are conceptually aligned with emerging AI and digital pathology literature, but participants emphasized that implementation must be safe, validated, auditable, interoperable, and integrated into existing workflows [19,20,31,32]. The feasibility, safety, effectiveness, cost-effectiveness, and health-system resource implications of these participant-proposed applications were not evaluated in this study and require prospective evaluation. Participants’ generally positive attitudes toward selected AI-enabled workflow supports should not be interpreted as evidence of comprehensive institutional readiness for implementation. This study assessed clinician perceptions of potential applications and associated safety considerations rather than technical, organizational, or operational readiness using a validated framework.

Implementation of AI within CRC NGS workflows also raises important privacy, governance, and safety considerations. Because these tools may process pathology, molecular, and EHR data containing protected health information, appropriate data-security safeguards, access controls, and privacy oversight are required. AI performance may also vary across institutions because of differences in patient populations, laboratory practices, data availability, and clinical workflows. Responsible implementation therefore requires local validation, bias assessment, auditability, clear accountability and ongoing monitoring for performance degradation or model drift [23,33,34].

Related AI applications have undergone technical validation or early clinical evaluation in oncology settings, although these studies do not establish the effectiveness of AI-enabled workflow support within the CRC NGS pathways examined here. For example, deep-learning models can infer mismatch-repair and microsatellite instability status directly from routine hematoxylin and eosin-stained slides, offering a rapid, low-cost pre-screen that could help prioritize confirmatory molecular testing and flag potentially eligible CRC cases earlier in the pathway [35,36]. Beyond molecular phenotype prediction, deep-learning systems have also been developed to detect colorectal adenocarcinoma, grade dysplasia, and classify colorectal tissue from whole-slide histopathology images, with retrospective studies reporting high diagnostic performance [37,38]. However, performance may vary across external datasets, and differences in training populations, slide preparation, scanner platforms, and limited prospective clinical validation remain important barriers to generalizability and routine implementation [39]. Automated, genomically driven clinical trial-matching platforms have similarly been deployed within enterprise sequencing and EHR systems to connect molecularly profiled patients to relevant trials in near real time [40]. Collectively, these studies demonstrate the potential of AI across pathology and molecular workflows, while emphasizing the need for external and prospective validation before routine clinical adoption.

Importantly, participants did not view AI as a replacement for clinical judgment. Instead, their responses support a human-in-the-loop model in which digital or AI-enabled tools improve the reliability of routine workflow tasks while clinicians remain accountable for eligibility decisions, interpretation, and treatment action. In the near term, the most feasible applications may be bounded workflow supports, such as flagging potentially eligible cases, identifying missing or overdue orders, and surfacing completed results, rather than autonomous interpretation or treatment recommendation.

This study has several strengths, including its multi-institutional design across Ontario, inclusion of both medical oncologists and pathologists, and use of qualitative methods to explain the mechanisms underlying delays that are often captured only as turnaround-time metrics.

Several limitations should be acknowledged. The sample was predominantly academic and Ontario-based, with limited representation from community centres, which may limit the transferability of the findings to other practice settings. Accordingly, the findings should be interpreted primarily as reflecting perspectives from the participating Ontario centres; community and send-out testing experiences were represented but were not sufficiently sampled to support subgroup-specific conclusions. Findings reflect clinician perceptions rather than directly measured testing rates, turnaround times, or patient outcomes. We did not interview several stakeholder groups implicated in the pathway, including surgeons, gastroenterologists, laboratory technologists, administrators, or patients. In addition, 22 of the 59 invited clinicians participated, representing a participation rate of approximately 37%. This may have introduced selection and non-response bias, as clinicians with greater interest in NGS workflows, quality improvement, or AI integration may have been more likely to participate. Consequently, the perspectives of non-participants may differ from those represented in this study, which may limit the transferability of the findings. Finally, the study was conducted within a Canadian publicly funded context and thus barriers and feasible interventions may differ in settings with different funding structures, laboratory networks, or EHR infrastructure.

Future work should quantify the frequency and impact of the barriers identified here, incorporate broader stakeholder perspectives, and prospectively evaluate interventions such as reflex-testing protocols, closed-loop tracking dashboards, structured reporting templates, and AI-enabled workflow supports, including their accuracy, safety, feasibility, workflow impact, acceptability, and resource implications.

## 5. Conclusions

CRC NGS pathways are vulnerable to delay and omission across biopsy and eligibility identification, test initiation, tissue handling and laboratory processing, result tracking and routing, reporting, and oncology decision-making. Across institutions, actionable gaps included inconsistent reflex-testing processes, unclear ownership of pathway steps, absence of closed-loop result tracking and routing, variable report usability, and fragmented digital infrastructure. Addressing these gaps will require pathway-level quality improvement rather than isolated changes to laboratory turnaround time alone, including standardized stage-appropriate reflex criteria and workflows, clear accountability, reliable result tracking and routing to the responsible medical oncology team, standardized molecular reporting, and stronger interoperable EHR integration. Clinicians expressed generally positive views toward digital and AI-enabled tools to support these improvements, provided such tools are safe, validated, integrated into existing workflows, and implemented with human-in-the-loop oversight. Together, findings from our study may help inform future QI initiatives and support national organizations in advancing improvements in molecular testing pathways, while further work is needed to determine their transferability to community settings and other Canadian jurisdictions.

## Figures and Tables

**Table 1:** Participant Characteristics.

Characteristic	Category	*n* (%)
Professional role	Medical oncologist	9 (40.9)
	Pathologist	13 (59.1)
Sex	Female	9 (40.9)
	Male	13 (59.1)
Institution Type	Academic	18 (81.8)
	Community	4 (18.2)
Years in practice	<5	5 (22.7)
	5–14	9 (40.9)
	≥15	8 (36.4)

**Table 2:** Quality gaps, consequences, and suggested quality improvement strategies across the CRC NGS Pathway.

Pathway Step	Identified Quality Gap(s)	Consequence(s)	Potential Participant-Proposed Quality Improvement Intervention(s)
Diagnosis and eligibility identification	Stage-limited NGS eligibility often unclear at pathology review, creating reliance on upstream clinicians to flag eligible cases	Delayed eligibility recognition and delayed test initiation	Create automatic NGS triggers or order sets for upstream clinicians Embed NGS initiation prompts into gastroenterology, surgical, radiology, and pathology workflows Define clear responsibility for flagging eligible cases Consider expanding funded CRC molecular tests beyond advanced disease as new stage-specific indications emerge
Tissue handling and NGS processing	Pathology staffing and support personnel constraints Laboratory equipment and machine processing often at capacity due to rising NGS volumes Limited/no in-house NGS capability at community centres, requiring send-out testing and additional turnaround time	Variable/delayed turnaround time	Recruit and retain pathology staff Improve laboratory infrastructure (e.g., point-of-care testing, additional laboratory equipment to increase capacity to run NGS) Improve community hospital NGS capacity when feasible Standardize tissue-handling and dissection workflow Create reliable send-out tracking processes
Result tracking and routing	Limited automated systems or supported personnel for NGS tracking in most centres External sent-out results may not reliably reach the oncology team Results may return to the ordering gastroenterology endoscopist or surgeon rather than the treating medical oncologist Some NGS results are faxed or scanned rather than automatically integrated into the EHR	NGS result reporting may be missed or delayed due to lack of tracking, no way to identify missed cases Participant-reported longer NGS turnaround times, particularly for community centres Medical oncologists may need to repeatedly contact pathology teams to clarify NGS status	Create closed-loop automated tracking dashboards with overdue-result alerts Improve EHR functionality to display real-time NGS status and automatic result updates, potentially supported by AI or other emerging digital technologies
Result reporting	Reports are lengthy, fragmented, addendum-heavy, or difficult to interpret Some NGS reports lack clinically relevant interpretation	Actionable NGS findings may be missed or applied incorrectly	Standardize molecular report format Prioritize actionable NGS findings at the top of the report Include clear variant tiering and clinically relevant interpretation, including therapeutic options and clinical trial matching where applicable
Digital infrastructure, EHR integration, and clinician perspectives on AI integration	Different EHR systems across centres with poor interoperability and fragmented result visibility Ongoing reliance on paper, fax, and manually scanned NGS reports for some centres Minimal AI integration or utilization within current NGS workflows	Missed or delayed NGS results, leading to delayed reporting and potentially duplicate NGS testing Delayed access to completed NGS reports Manual-intensive and time-consuming workflows for clinicians and support staff	Enable automatic upload of NGS reports into EHR through EHR optimization and/or AI-enabled integration Create an integrated molecular result table within EHR including NGS, MMR/MSI status, and subsequent report updates Improve interoperability between hospital EHRs, pathology systems, and external molecular laboratories Standardize electronic NGS requisition with required clinical information, including staging and NGS testing indication Use AI or digital tools to flag eligible cases, identify missing NGS orders, and monitor overdue results

Note: The strategies presented in Table 2 were proposed by interview participants and were not evaluated for feasibility, effectiveness, cost-effectiveness, laboratory capacity, or health-system resource implications. They should be considered potential strategies requiring prospective evaluation rather than established recommendations.

## Data Availability

The raw data supporting the conclusions of this article will be made available by the authors on request.

## Abbreviations

The following abbreviations are used in this manuscript:

AI	Artificial Intelligence
BRAF	B-Raf Proto-Oncogene, Serine/Threonine Kinase
BRCA	Breast Cancer Gene (BRCA1/BRCA2)
CCO	Cancer Care Ontario
CRC	Colorectal Cancer
EHR	Electronic Health Record
ERBB2	Erb-B2 Receptor Tyrosine Kinase 2
HER2	Human Epidermal Growth Factor Receptor 2
HSREB	Health Sciences Research Ethics Board
IHC	Immunohistochemistry
KRAS	Kirsten Rat Sarcoma Viral Oncogene Homolog
MMR	Mismatch Repair
MSI	Microsatellite Instability
NGS	Next-Generation Sequencing
NRAS	Neuroblastoma RAS Viral Oncogene Homolog
PHI	Protected Health Information
PIK3CA	Phosphatidylinositol-4,5-Bisphosphate 3-Kinase Catalytic Subunit Alpha
PTEN	Phosphatase and Tensin Homolog
QI	Quality Improvement
RAS	Rat Sarcoma Viral Oncogene Homolog
VUS	Variant of Uncertain Significance

## Appendix A

**Notes:** Quotes from interviews highlighting identified themes. Quotations presented in the main text and Appendix A Table A1 have been lightly edited to remove non-contributory words for readability; wording and meaning are otherwise preserved.

**Interview Guide**

1. Can you briefly describe your role and how you are involved in NGS testing for colorectal cancer? Probe: At what points in the patient journey do you interact with molecular testing?
2. How do you currently decide when NGS testing should be performed for patients with colorectal cancer? Probe: Are indications clear and standardized, or variable across providers?
3. From your perspective, are NGS tests ordered at the optimal time in the care pathway Probe: Early diagnosis vs. metastatic setting; pre- vs. post-surgery.
4. Are there situations where NGS testing is delayed, omitted, or repeated unnecessarily? Why?
5. How do funding structures or test coverage influence when and how NGS testing is ordered for colorectal cancer Probe: Public funding criteria, institutional policies, or access to external programs.
6. Are there situations where funding limitations delay or prevent NGS testing, even when it may be clinically useful?
7. How often does tissue adequacy or availability limit the ability to complete NGS testing?
  Probe: Biopsy size, fixation, prior testing, surgical vs. endoscopic samples.
8. How well is communication coordinated between surgery, pathology, and oncology around tissue stewardship?
9. In your experience, are expectations around tissue use and prioritization aligned across specialties?
10. Can you walk me through the NGS workflow from your perspective, from test initiation to result reporting?
11. Where do you see the most common bottlenecks or delays in the NGS pathway? Probe: Ordering, send-out, sequencing, interpretation, reporting.
  Follow up: To your knowledge, are there challenges in finding enough qualified staff (technologists, geneticists, molecular pathologists) at your institution to meet workload demands?
12. Is there a clear sense of who is responsible for tracking NGS tests once they are ordered?
13. Who signs out CRC molecular at your institution? Probe: PhD Molecular geneticist? MD fellowship-trained? molecular pathologist? MD non-fellowship trained pathologist with targeted training? Combination of people with different credentials
14. How do delays in NGS results affect clinical decision-making or treatment planning?
15. Are NGS reports clear, timely, and clinically actionable from your perspective? Probe: Clarity of variants, interpretation, therapeutic relevance.
  Follow up to ask oncologists: Do you prefer molecular reports to include commentary on therapeutic implications or do you find them to be a distraction? If yes, how detailed do you prefer such comments to be?
16. Have you ever encountered situations where a report was misinterpreted or unclear, and where that uncertainty influenced, or potentially changed, treatment recommendations?
17. How confident do you feel in integrating NGS results into treatment decisions? Probe: Role of molecular tumour boards or expert consultation.
18. Are there results that come back too late to influence care meaningfully?
19. How well do different specialties communicate around NGS testing for colorectal cancer?
20. Are there gaps in handoffs or ownership across oncology, pathology, and surgery?
21. How does the current electronic health record support or hinder NGS ordering, tracking, and result review?
22. Do you think all eligible colorectal cancer patients are receiving NGS testing equitably? Probe: Differences by disease stage, referral source, or care pathway.
23. If you could change one aspect of the NGS pathway to improve timeliness or quality, what would it be?
24. What would an ideal NGS testing pathway for colorectal cancer look like in your setting?
25. Is there anything else about NGS testing for colorectal cancer that you think is important but often overlooked?

**Table A1:** Quotes from interviews highlighting identified themes.

Subtheme	Illustrative Quotation	Participant
**Theme 1. Clinical indications, patient identification, and test ordering**		
1.1 Upstream clinician role in flagging cases (*n* = 18)	*“I would prefer the clinicians to tell me upfront whether they want NGS testing or not. Not me trying to search all sorts of information to know whether I’m to audit or not. That’s a thing, … we spend a lot of time, which can be easily saved right now, because a lot of our clinicians, they even for surgery past cases, they don’t give us any clinical information.”*	[Pathologist, academic centre]
	*“Ideally, the pathology for colorectal, the surgeon or the endoscopist, right when they’re doing the endoscopy, on the pathology report, they say suspicious for Stage Three colorectal cancer, NGS is required, and then that gets to the pathologist, and that automatically, as soon as they confirm adenocarcinoma on the first stain, they send off a block for NGS.”*	[Medical Oncologist, community centre]
1.2 Reflex vs. clinician-requested testing (*n* = 22)	*“For any metastatic colorectal cancers or advanced colorectal cancers, like NGS is like a reflex testing, right? But in our institution, most of time, yes, sometimes no, it’s not being very, very consistent because of sometimes… the restraint in the lab resources and limits in the like staff, like a time. So a lot of times we would be ordering by request.”*	[Pathologist, academic centre]
	*“The NGS is ordered reflexively if the pathologist at the time that they’re reading the film knows it’s a stage 3 or stage 4 patient. If it’s a colon cancer biopsy, they often won’t know that.”*	[Medical Oncologist, Academic Centre]
1.3 Uncertainty when stage is unknown at biopsy (*n* = 14)	*“On biopsies, it’s a little bit … less consistent because of both variable amounts of information available at the time of biopsy, and if I were being honest, variable tendencies of pathologists to scour into the available information.”*	[Pathologist, academic centre]
	*“So the problem is people who have a biopsy in the community, and then no one knows what stage they are, right, because they just have a colonoscopy, and then they subsequently get imaging, and then they realize they have advanced disease that’s metastatic, and then they show up at the cancer center and they don’t have their RAS testing.”*	[Pathologist, academic centre]
1.4 Evolving indications beyond metastatic disease (*n* = 15)	*“Then this, like New England Journal paper came out, indicating that aspirin maybe may help prevent recurrence and early, earlier stage since that article came out, we’ve been getting requests to do it on stage two and stage one disease for that reason. Presumably, we haven’t moved to doing that reflex yet, but we may shift to doing it because of that.”*	[Pathologist, community centre]
	*“So with ALASCCA now for stage two, especially the younger patients who have a higher chance of having a PTEN or PIK3CA mutation, there will be more communication between surgery and medical oncology about getting this testing done.”*	[Medical Oncologist, academic centre]
1.5 Administrative/manual burden of test initiation (*n* = 7)	*“I guess administrative role is that I probably 50% of the time have to initiate NGS testing on these colorectal patients because it hasn’t been done, because maybe it wasn’t flagged by either the endoscopist or the pathologist that it was a metastatic case. So I’d say half the time I have to trigger it.”*	[Medical Oncologist, academic centre]
	*“Sometimes you think you’ve ordered it and you forget it. So those are all practical reasons why it might be missed”*	[Pathologist, community centre]
1.6 Funding coverage shaping ordering decisions (*n* = 17)	*“It’s costly to run the NGS, both in time and resources, so we really look for CCO funding to be in place to make sure we’re getting reimbursed in terms of doing this testing. So that’s the, I guess, the major limiting factor in that sense, that being said at the end of the day, it’s a patient. And so if we get a request outside of those parameters that an oncologist or someone wants that testing done, we just kind of go ahead and do it and absorb it.”*	[Pathologist, community centre]
	*“Not testing earlier stage just because it’s not officially one of the CCO funded indications, and just because our lab… the volumes are very high, and so we need to limit as much as possible, so we’re not testing any additional indications.”*	[Pathologist, academic centre]
**Theme 2. Tissue, pathology, and molecular laboratory workflow**		
2.1 Send-out vs. in-house testing (*n* = 17)	*“We’re a small operation, and we’re trying to figure out how to bring it back, hopefully in the near future, because sending it out is a struggle.”*	[Pathologist, academic centre]
	*“It was faster when it was in-house and then you had the molecular pathologist who you could message directly, which was very nice.”*	[Medical Oncologist, academic centre]
2.2 Laboratory capacity, staffing, and infrastructure (*n* = 17)	*“I guess our big delay is due to staffing in our molecular lab, I think probably, most places around the country, is pretty understaffed, so tests can take a long time, certainly more than a month, usually, and sometimes it could be up to eight weeks or so”*	[Pathologist, academic centre]
	*“So it’s easier to get more equipment. If you have a high volume, it’s actually easier to get more equipment, more reagents. But then the CCO funding doesn’t cover the personnel.”*	[Pathologist, academic centre]
2.3 Bottlenecks and turnaround-time variability (*n* = 22)	*“The sequencing and the library preparation instruments frequently have issues, and so they frequently need to be repaired. There’s also frequent reagent issues, like the quality control of the reagents. and the company that supplies our reagents for the NGS testing is not really up to snuff, and so there’s frequent reagent problems with that cause runs that need to be repeated. So those are big causes of delays as well.”*	[Pathologist, academic centre]
	*“One of the biggest delays happens at the pathologist side. So you have the molecular results, it’s just getting that somebody’s going to go back and revise a report. And that’s just the other one with workloads.”*	[Pathologist, academic centre]
2.4 Varying tissue-dissection approaches across sites (*n* = 7)	*“So we don’t circle tumour or micro dissect, as other labs do with a we actually just scrape the entire slide so they’ll cut, I think it’s 10 additional tissue sections from the block. They’ll scrape that tissue and run it through the NGS protocol in terms of the amplification. Once that’s done, which is actually quite quick we do, runs pretty much every day, unless there’s something weird going on. So I’ll typically have a result back, I would say, in like two to three business days.”*	[Pathologist, community centre]
	*“We do a macro dissection to concentrate the area of tumour. Then within the lab, they extract that area. We are currently partially using some liquid handlers to handle with some of the DNA extraction that goes through a QA process.”*	[Pathologist, academic centre]
**Theme 3. Interdisciplinary communication and test tracking**		
3.1 Informal communication pathways (*n* = 20)	*“I email the person I know in pathology, or I contact that person, and they tell us where it is, and essentially the answer is always, is it was sent on this date? We don’t have a result. And so then I say, Can you please tell them to make it urgent, and if it’s truly, truly urgent, then they just email, I just email data directly. And he usually tells me where it is and when they would expect it was.”*	[Medical Oncologist, community centre]
	*“So if I ask someone from pathology to do something or to clarify, they will, and we can pick up the phone or text them or whatever. So I think our interdisciplinary collaboration, although unofficial, and the terms of being done through text and whatever, it’s actually pretty good, right?”*	[Medical Oncologist, community centre]
3.2 Unclear ownership of pathway steps (*n* = 12)	*“I mean, one of the things that we’ve been discussing and we’re hoping to actually sit down with all the stakeholders is that initiation of testing in an earlier line and how do we provide the intrinsic motivation of teams like surgery to actually care because very rightfully one of the counters will be well, we don’t prescribe these drugs, it doesn’t really matter to us”*	[Medical Oncologist, academic centre]
	*“There’s gaps. For example, right now, if you put all of us in front of a computer and said, find out who ordered this and why, all of us would be like, okay, you know, we wouldn’t know.”*	[Medical Oncologist, academic centre]
3.3 Tracking pending results: manual, individualized methods (*n* = 20)	*“There’s absolutely no way to track where the testing is at.”*	[Medical Oncologist, academic centre]
	*“I keep an area in my office for cases that are pending some sort of additional testing. And so periodically I go through it. So if, for example, I don’t receive NGS results back in a week without the abnormal for my center. So then if I have a case that’s pending NGS for more than a week, I’ll reach out to our technical team in terms of following up”*	[Pathologist, community centre]
3.4 Need for closed-loop confirmation and alerts (*n* = 11)	*“I think ideally we want flags, or if an order is being put in and the result has not come out, within a set parameter, we get some sort of alert, like if you put in an NGS result and there’s nothing after a week, something is flagged for the pathologist or technologist to take a look at that case because that exceeds the parameter”*	[Pathologist, community centre]
	*“Once the test is ordered, there’s no good consistent system to see where that test is at. So you could ask for the test, but when we go into our EHR, there’s nowhere where we can find out if the test has been sent.”*	[Medical Oncologist, academic centre]
**Theme 4. Reporting, interpretability, and clinical integration**		
4.1 Variability across labs in which alterations are reported (*n* = 15)	*“Our MMR status is immunohistochemistry, so technically not on our NGS. So the RAS, BRAF, PIK 3CA are the top ones that we’re looking for.”*	[Medical Oncologist, academic centre]
	*“So from the NGS, we are looking for extended RAS and RAF testing. You know, BRAF testing is very important, of course. I mean, the panel is broader than that it does look at ERBB2. We don’t have access to IHC for HER2, so we use ERBB2 as a screening… PIK3CA testing is also part of that. They do PTEN. So that is our panel.”*	[Medical Oncologist, academic centre]
4.2 Report clarity and usability (*n* = 18)	*“The way they have now summarized the reports, I think is quite clear in terms of if it’s actionable or not, tier one or tier 2”*	[Medical Oncologist, academic centre]
	*“In my opinion, you know, it’s not they’re ready, they’re just not easy to interpret, right? And I can tell you, it’s very possible that something can easily be missed, right? An actual mutation can be missed because it’s just buried under multiple levels of paper, right?”*	[Medical Oncologist, community centre]
4.3 Variable need for interpretive support (*n* = 11)	*“So we have an established molecular tumour board. It has been running now for maybe a year or two. And I have occasionally presented cases of things I’m uncertain about. So the patient who had a BRCA mutation and their colorectal cancer, I did present there because I wanted a bit more understanding about what this meant.”*	[Medical Oncologist, community centre]
	*“I don’t tend to get very specific questions about a specific finding. They’re usually not like, Hey, you have this finding on the report. I don’t understand that.”*	[Pathologist, academic centre]
4.4 Timing of report relative to treatment decision (*n* = 14)	*“But the majority of patients, unfortunately, still probably about 70% at the time of first consult, our NGS is not ready. We’re the ones asking for it and initiating it. And then and we’re the ones that have to make a additive chemo decision because we don’t have it on the chart, or a complete treatment decision because we don’t have it on the chart at the first consult.”*	[Medical Oncologist, academic centre]
	*“It would be very nice to be able to have NGS testing by the time of consultation or especially by the time of first treatment, but oftentimes it’s not. So we’ll get MMR testing, but NGS testing oftentimes won’t come back until patients have been on cycle 2 or cycle 3. It just takes that long.”*	[Medical Oncologist, academic centre]
**Theme 5. EHR integration, digital infrastructure, and clinician perspectives on AI integration**		
5.1 Paper, fax, and manual data-entry persistence (*n* = 10)	*“I don’t think people realize how much in the Stone Age we are in terms of fax machines and paper forms and just how these processes are still so inefficient”*	[Medical Oncologist, academic centre]
	*“It’s not directly uploaded to our system when that testing has been done. We’re relying on a fax from the outside institution and then that gets uploaded into the EHR.”*	[Medical Oncologist, academic centre]
5.2 Fragmented EHR visibility (*n* = 18)	*“I think the biggest problem is technological right now. It’s the EHRs that aren’t telling us where the NGS is and how they’re sending it. It’s mostly technological process things. It’s not the people. It’s a tech issue”*	[Medical Oncologist, academic centre]
	*“In our current system with Cerner, there is the specimens, because in molecular they’re working in a different system called Shire, the histology clerks need to do more paperwork at the beginning before it goes up to molecular, they need to print off a patient sticker that has all of their identifiers,* etc. *And then I think, once it gets to molecular, they need to re access it, so it adds work for them in order for it to get into Shire.”*	[Pathologist, academic centre]
5.3 Limited interoperability across institutions (*n* = 17)	*“Because our electronic health records are very disjointed, so I can’t see it obviously that it’s been done. I just do it, even if it may have been done at another center, and I just can’t see those results.”*	[Pathologist, community centre]
	*“Connecting Ontario? I don’t know, it seems like a lot of institutions are unconnected in Ontario, but there’s still reports missing. So I have sent like cases … for consultation. But then I got the consult letter in my hand, but then when I check on connecting ontario, there’s nothing, so it’s very clearly, it’s not uploaded, or missed”*	[Pathologist, academic centre]
5.4 Digital tracking, alerts, and result notification (*n* = 9)	*“I think we need some other system where, you know, even, I don’t know if the pathologist somehow can, they say sometimes that NGS has been ordered, they don’t always say that though. So right from the beginning I find it very unclear.”*	[Medical Oncologist, academic centre]
	*“When I think of AI, I think you know first… tracking communication, and then maybe an interpretation… So I don’t know if that’s in our system or outside of our system, but a platform that tracks things, and a platform that provides things updates and when results are available. So really, I guess what’s missing our EHR”*	[Medical Oncologist, academic centre]
5.5 AI for triage, case identification, and reflex initiation (*n* = 20)	*“So patients that are going through the system, if we had an AI agent that looks to see, you know, which patients should have had NGS, where that NGS is at, that I think would help.”*	[Pathologist, academic centre]
	*“My first thought would be, AI would recommend when reflex testing is appropriate. So if AI would scan the clinical history and the pathology report and then flag if case like this should be sent for these reflex tests, that would be fantastic if that flag appeared in our lab information system.”*	[Pathologist, community centre]
5.6 Trust, validation, and human oversight of AI (*n* = 18)	*“But the limitation right now is certainly around PHI and where that information lives and is it safe and how trustworthy is something doing that interpretation, like what’s the fidelity and accuracy of it?”*	[Medical Oncologist, academic centre]
	*“I’d be open to implementing it. I’m just saying that I think part of our policy would be to have some check in, whether it’s every maybe every three months or every six months or once a year, I don’t know, but it would be foolish to just sort of trust it indefinitely and not do audits”*	[Pathologist, community centre]
